# Experimental simulations of volcanic ash resuspension by wind under the effects of atmospheric humidity

**DOI:** 10.1038/s41598-018-32807-2

**Published:** 2018-09-28

**Authors:** E. Del Bello, J. Taddeucci, J. P. Merrison, S. Alois, J. J. Iversen, P. Scarlato

**Affiliations:** 1 Istituto Nazionale di Geofisica e Vulcanologia, Sezione di Roma 1, Via di Vigna Murata 605, 00143 Roma, Italy; 2 Aarhus University, Department of Physics and Astronomy, Ny Munkegade 120, 8000 Aarhus C, Denmark

## Abstract

Ash deposited during volcanic eruptions can be resuspended by wind and become hazardous for health and infrastructure hours to decades after an eruption. Accurate resuspension forecasting requires accurate modelling of the threshold friction velocity of the volcanic particles (*U*_th_*), which is the key parameter controlling volcanic ash detachment by wind. Using an environmental wind tunnel facility this study provides much needed experimental data on volcanic particle resuspension, with the first systematic parameterization of *U*_th_* for ash from the regions Campi Flegrei in Italy and also Eyjafjallajökull in Iceland. In this study atmospheric relative humidity (and related ash moisture content) was systematically varied, from <10% to >90%, which in the case of the Eyjafjallajökull fine ash (<63 μm) produced a twofold increase in *U*_th_*. Using the Campi Flegrei fine ash (<63 μm) an increase in *U*_th_* of only around a factor of 1.5 was observed. Reasonable agreement with force balance resuspension models was seen, which implied an increase in interparticle adhesion force of up to a factor of six due to high humidity. Our results imply that, contrary to dry conditions, one single modelling scheme may not satisfy the resuspension of volcanic ash from different eruptions under wet conditions.

## Introduction

**Figure 1:**
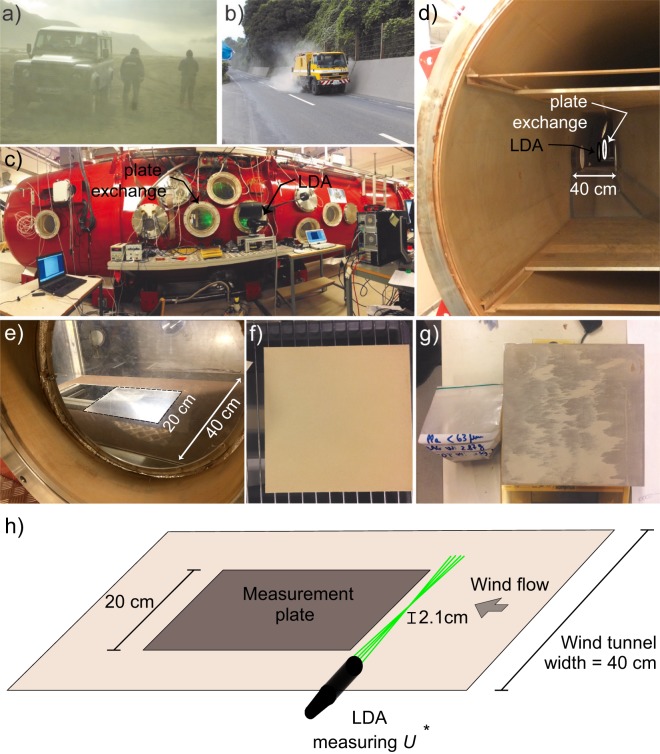
Examples of volcanic ash resuspension by wind around the Eyjafjallajokull volcano (Iceland) in May 2010 (**a**), and by road cleaning at Sakurajima volcano (Japan) in July 2013 (**b**). External view of the environmental chamber at Aarhus University showing Laser Doppler Anemometry (LDA) measurements (**c**). Internal view showing the test section of the wind tunnel (40 cm in width). The LDA and sample exchange windows are indicated (**d**). View of the test section from the LDA window, including the position (contoured) of the 20 × 20 cm plate (**e**). The experimental sample plate loaded with ash before an experimental run (**f**), and during an intermediate removal step (**g**). Schematic of the wind tunnel orientation used in this study as well as the LDA position for shear stress measurement (located 2.1 cm above the floor of the test section) (**h**). All Photographs and drawings were created by the contributing authors.

Volcanic ash affects life and infrastructures both during and after explosive volcanic eruptions. In fact, as well as the eruptive cycle – from injection, through atmospheric transport, to deposition on the ground –, one of the main sources of hazard related to the presence of volcanic ash is the wind-induced resuspension of particles^1–5^. Resuspended ash clouds travel at low atmospheric altitudes causing multifaceted hazards at variable time- and space-scales, as demonstrated by recent eruptions of variable magnitude and style (e.g., Eyjafjallajökull, Iceland 2010^2^, Cordón Caulle, Chile 2011^1^, Ontake, Japan 2014^6^). Resuspension events after the 1991 eruption of Hudson volcano (Chile), caused agricultural, environmental, and health impacts that prompted the evacuation of rural communities months after the eruption^7^. Months after the Cordón Caulle eruption, nation-scale air and road traffic in Argentina suffered from a large resuspension event^8^. Even more than a century after a large eruption, airport operations at the Kodiak Airport (Alaska) have been disrupted at times of drought due to the sedimentation of millimetric loads of resuspended ash from the Katmai region^9^. In the city of Catania (Italy), resuspension of ash erupted by the nearby Etna volcano frequently causes temporary increases the concentration of hazardous, breathable fine particulate matter (PM10)^10^. We directly witnessed ash resuspension in different scenarios, during the 2010 eruption at Eyjafjallajökull and during the repeated Vulcanian activity of Sakurajima volcano (Japan) in 2013 (Fig. 1a,b).

In recent years, several numerical simulation schemes, also hazard-oriented, have been developed and applied to volcanic ash resuspension events^6,8,11–14^. Critical to model the resuspension of particles by wind is knowledge of the minimum wind velocity required to initiate the detachment of particles laying on the ground, which is expressed by the threshold friction velocity parameter (*U*_th_*) through the relation ${\tau }_{th}=\,{\rho }_{f}{U}_{th}^{\ast \,2}$, where ${\tau }_{th}$ is the threshold flow shear stress and *ρ*_*f*_ is the atmospheric fluid density^15^. Current simulations of ash resuspension events lack direct information on the *U*_th_* values appropriate to volcanic ash particles under the relevant field conditions. Instead, these simulations are based on wind tunnel experiments on soils, sand, and synthetic particles^16,17^. These experiments, however, focus on mostly spherical particles with constant density, while volcanic ash particles have strongly size-dependent physical properties and typically include internal voids (vesicles) that control their density, surface area, shape, and water retention capability^18–20^.

*U*_th_* is defined by the balance between gravitational/adhesion forces and lift/torque forces acting on a particle at rest on a surface and subject to wind^21^. Several wind tunnel studies have assessed the dependency of *U*_th_* on the size, density, and shape of dry, non-volcanic particles^15–17,21–25^. Environmental humidity and moisture content of the deposit also play a role, as demonstrated by studies on wet, non-volcanic particles showing that the presence of water increases inter-particle cohesion, making particles less prone to resuspension^26–29^. Wind tunnel studies on volcanic particles are very limited and the effect of humidity had not been parameterised. First attempts to quantify *U*_th_* for volcanic ash from Mount St. Helens showed that loose, dry ash is far easier to remobilise than ash consolidated by wetting and drying processes^4^. Later studies on volcanic materials focused on the detachment mechanism - saltation or rolling - showing the dependency of these processes on volcanic particle grain size, shape and density^30–32^.

In the study presented here the first systematic parameterization of *U*_th_* of natural volcanic ash particles as a function of their grain size and of environmental humidity (and the related moisture content of the ash) has been carried out. In addition, we investigated volcanic ash samples from two different volcanic eruptions, showing the role of particle variability on their resuspension behaviour.

## Materials and Methods

Resuspension experiments have been carried out using an environmentally controlled recirculating wind tunnel facility at Aarhus University^22,33^ (Fig. 1c–h), with a chamber volume of around 35 m^3^ and a wind tunnel section of >4 m in length. Here the atmosphere (pressure and composition) could be controlled as well as generating a controlled wind flow. Reproducible wind flow was achieved by controlling the rotation rate of the fan system within the environmental wind tunnel. Wind speeds of up to 20 m/s were measured during this investigation. Upwind of the sample section was a 1.5 m long wind tunnel section in which the surface roughness was controlled using sand (sized 125–250 µm) adhered to the surface. Although these experiments were performed at ambient (1 bar) pressure, the relative humidity (RH) was controlled by evacuating the environmental chamber and either refilling it with dry air (extracted through a low temperature moisture trap held at −20 °C to −40 °C) or by the addition of water vapour into the chamber (extracted from a high temperature water container). The relative humidity within the chamber was monitored using a commercial sensor (Honeywell HIH-4602-C) as well as a portable hygro-thermometer (AMEC AM9651, accuracy ±3% RH). The effect of changing relative humidity on the atmospheric fluid density inside the chamber is small compared to typical error bars, the effect on gas mass density being <1.6% and the effect on the measured friction velocity U* being <0.8%. Temperature and pressure were also monitored. During the experiments the chamber was hermetically closed. Samples were exchanged through an access port.

The friction velocity U* and therefore also the surface shear stress ($\tau ={\rho }_{f}{U}^{\ast 2}$, where ${\rho }_{f}$ = 1.2 kg/m^3^) generated by the wind flow was determined using a 2D Laser Doppler Anemometer (www.dantecdynamics.com) which was capable of simultaneously measuring the vertical and horizontal wind flow velocity within a small (<1 mm^3^) volume. Measurements were performed 2.1 cm above the surface upwind of the sample for the various wind speed values used (Fig. 1h). From the variance in these wind velocities it was possible to determine the friction velocity ${U}^{\ast 2}=0.47\times (\sqrt{\overline{U{\text{'}}^{2}}}\sqrt{\overline{W{\text{'}}^{2}}})$^34^ at any wind speed, where $\overline{U\text{'}}$ and $\overline{W\text{'}}$ are the mean wind velocity fluctuation in the horizontal and in the vertical direction, respectively. From linear regression of wind speed and friction velocity we obtained a Round Mean Squared Error of U* of 0.08 m/s. Using LDA, the turbulence level (standard deviation) of the free flow in the wind tunnel section was measured to be around 11%. The flow uniformity across this channel was seen to be within this turbulence level (up to a few cm from the walls).

### Experimental procedure

To parameterise resuspension we considered the amount of particles detached from a granular surface covered by a homogeneous layer of particles per unit area and time. Each detachment experiment was executed as follows: ∼5 g of volcanic ash was sieved directly onto a 20 × 20 cm aluminium plate, precoated (glued) with a homogeneous, 1-mm-thick substrate of the same sieved ash. The plate was weighed, then inserted into the wind tunnel from an exchange window, and exposed to consecutive steps of increasing friction velocity starting from 0.1 m/s until maximum obtainable U* (about 0.6–1 m/s) or end of the removal. During each step the wind speed was kept constant for a period of 2 minutes, then the plate was extracted from the tunnel and weighed (ViBRA AJ series scale, precision 0.01 g) to obtain the removed mass (Fig. 1e–g).

Sample moisture was controlled by first oven drying the samples at 110 °C until totally dry, then leaving them in the wind tunnel for 12–15 hours at a fixed RH range (0–10%, 50–70%, 80–90%, and 90–100%), and finally performing the experiment at the same RH range. No visible liquid water was deposited on the test plate nor in the visible wind tunnel section during the experiments. We estimated the moisture content *w* (wt. %) of the samples with the maximum moisture (RH 90–100% experiments) by precision weighing (with a Sartorius model BP301S scale, precision 0.0001 g) of control samples that underwent the same dehydration-hydration routine as the experimental ones.

### Volcanic ash properties

**Figure 2:**
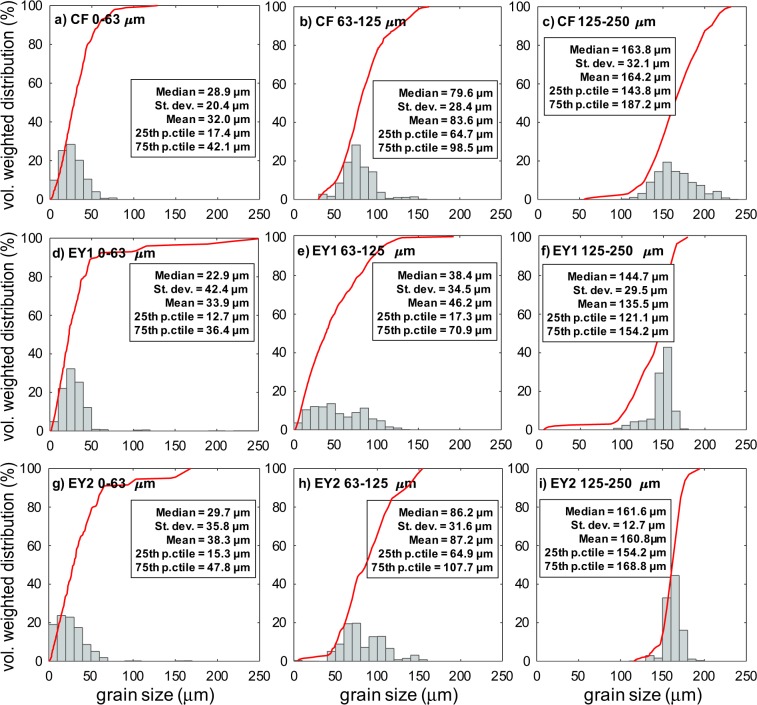
Grain size distribution of the Campi Flegrei-Pomici Principali (*CF*, **a**–**c**) and Eyjafjallajokull (*EY*, **d**–**i**) ash samples in the three size classes (0–63 μm, 63–125 μm, and 125–250 μm) used in wind tunnel detachment experiments. In red, the cumulative distribution curves, and in the text boxes the distribution parameters. Note the fines-enriched and broader distribution of the 63–125 μm *EY1* sample (**e**) with respect to the corresponding *EY2* one (**h**).

**Figure 3:**
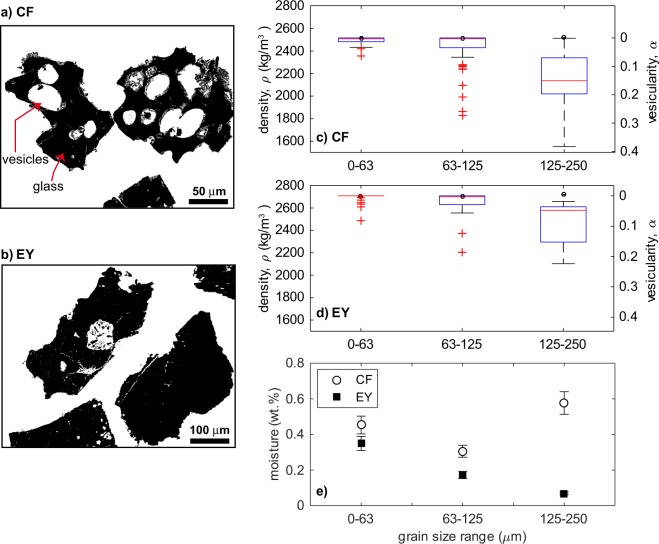
Properties of the *CF* and *EY* ash samples starting material. Selected Scanning Electron Microscope images of ash thin sections after gray scale thresholding, showing particles area (black) and internal vesicles (**a–b**). Note the more vesicular appearance of the *CF* particles (**a**) with respect to the *EY* ones (**b**). Box diagram showing the median value (red line), the 25^th^ and 75^th^ percentiles (box size), 99.3 percent of data (whiskers), and outliers (red crosses) for the distribution of density and 2-D vesicularity of the particles as a function of grain size class. Open circles represent the density of the solid fraction (**c–d**). Variation of the moisture content of samples at RH 100% as a function of the sample size class (**e**). Error bars in the moisture reflects the weighting error. Note the divergent trend for the coarsest size class.

As starting materials, we used volcanic ash from two fallout deposits: the phonolitic, ~10 ka old Campi Flegrei-Pomici Principali Layer A^35^ (*CF* samples), and the basaltic-trachyandesite^36^ products settling from the Eyjafjallajokull eruptive plume on 19 May 2010 (*EY* samples). After oven drying, the ash was sieved manually into three grain size classes: *d* < 63 µm; 63 < *d* < 125 μm, and 125 < *d* < 250 μm, where *d* is the particle diameter. A second batch of the *EY* sample was also sieved to investigate possible sample preparation and grain size distribution effects on resuspension. The granulometric distribution of the ash in each class was characterized by optical granulometry (LUMiReader Particle Size Analyser^37^) (Fig. 2). For each class, high-resolution images of loose particles and of thin sections of particles embedded in resin were captured by Scanning Electron Microscopy (JEOL JSM-6500F). From the thin section images and using the ImageJ analysis toolbox^38^ we measured the area of both particle cross-section and of all internal vesicles for a population of 50 ca. clasts per class. For each particle we also calculated the literature *F* and Ψ particle shape parameters^39,40^. *F* = (b + c)/2a, where a, b, and c are the longest, intermediate and shortest axes of the particle best-fitting ellipsoid, respectively. Ψ = Φ /χ, where Φ is sphericity (the surface area of the equivalent sphere divided by the surface area of the actual particle) and χ is circularity (the particle perimeter divided by the equivalent circumference of a prolate ellipsoid). The *F* and Ψ parameters are constant at 0.65 and 0.40 (where the two parameters would be equal to one for perfect spheres), respectively, with the only exception of Ψ = 0.60 for the 0–63 µm samples. The 2-D vesicularity *α* of each particle was computed by dividing the total area of the vesicles by the total area of a particle (Fig. 3a–d). We also measured the density of the solid fraction of the ash (i.e., without vesicles, *ρ*_*s*_) using a Helium pycnometer (Micrometrics AccuPyc II 1340), obtaining an almost constant *ρ*_*s*_ of 2515 ± 3 kg/m^3^ and 2708 ± 8 kg/m^3^ for *CF* and *EY*, respectively, regardless of the size class. The actual density of the particles (i.e., including vesicles, *ρ*) was computed as $\rho \,={\rho }_{s}(1-\alpha ),$ assuming that measured 2-D vesicularity is representative of the actual 3-D vesicularity of the particle. Obtained values of *ρ* decrease with increasing grain size from 2509 kg/m^3^ to 2139 kg/m^3^ (*CF*) and from 2692 kg/m^3^ to 2587 kg/m^3^ (*EY*). Maximum sample moisture content obtained for the experiments run at RH 90–100%, ranged between 0.06 and 0.58 wt.% (Fig. 3e).

## Results

**Figure 4:**
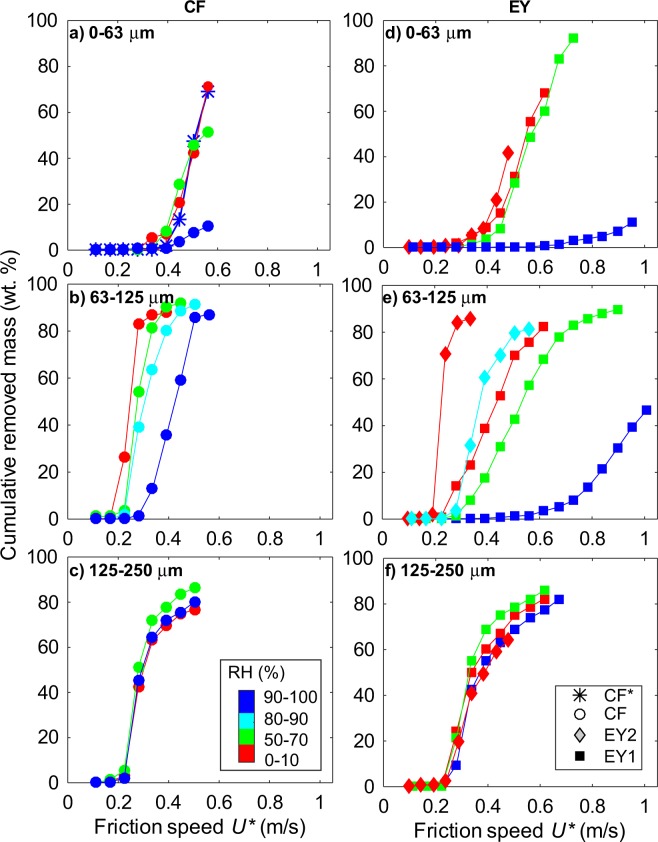
Removal curves for *CF* (**a–c**) and *EY* (**d–f**) ash samples of variable moisture content, sieved in the 0–63, 63–125 and 125–250 μm size classes. Each data point represents the cumulative mass of particles (error ± 0.3 wt. %) removed at the corresponding friction speed step (error ± 0.08 m/s), colour-coded by wind tunnel humidity range (RH). CF* indicate an experiment carried out with a dry sample (nominal moisture content 0 wt. %), in high humidity environment (RH 90–100%).

A total of 25 detachment experiments were carried out, covering two ash types (*CF*, *EY*) in three grain size classes (0–63, 63–125, 125–250 µm), and at up to four relative humidity ranges (0–10%, 50–70%, 80–90%, and 90–100%). For each experiment, a cumulative removal curve was obtained by plotting the mass percentage of removed ash (over the initial sample mass) at each incremental wind speed step (Fig. 4). Steeper curves characterise ash samples where starting conditions (sample grain size, moisture, and type) favour a more prompt detachment, generally occurring at lower friction speed values. At most, the curves reaches a plateau at ca. 90% of removed particles, some grains always remaining on the plate.

In the ‘dry’ (RH 0–10%) cases, removal curves are similar for the *CF* and *EY* samples. The only exception is in the *EY* 63–125 μm size class (Fig. 4e), where the fines-poor *EY2* sample behaves similarly to the *CF* one, while the fines-rich *EY1* sample largely deviates towards gentler curves, more similar to the *EY* 0–63 μm size class sample. Conversely, the *EY1* and *EY2* samples display quite similar curves in the other size classes.

Non-‘dry’ (RH > 10%) cases show that the effect of RH on detachment decreases with increasing grain size, becoming entirely negligible for the 125–250 μm size class. For the other size classes, the effect of humidity is much smaller in the intermediate (RH 50–90%) cases than in the ‘wet’ (RH 90–100%) ones. Comparison of the *EY1* and *CF* samples in the 0–63 μm size class, shows that in ‘wet’ (RH 90–100%) environments, the former is more difficult to remobilise than the latter. Comparison of the *EY2* and *CF* in the 63–125 μm size class and RH 80–90% humidity shows a less marked but similar trend of less mobility for the EY sample. As an additional test, a completely dry (i.e., kept in the oven until the experiment) *CF* 0–63 μm sample was run in the humid wind tunnel (RH 90–100%). The results (Fig. 4a) closely mimic those of the same sample at RH 0–10% conditions.

Experimentally, the threshold friction velocity *U*_th_* is determined from detecting initial movement of particles^21,25,27^. In this study threshold friction velocity values were calculated from the removal curves, by applying a smoothing spline piece-wise polynomial fit and taking the value of U* corresponding to 10% of the removed mass, marking an unequivocal rise of the curve at the onset of substantial removal.

**Figure 5:**
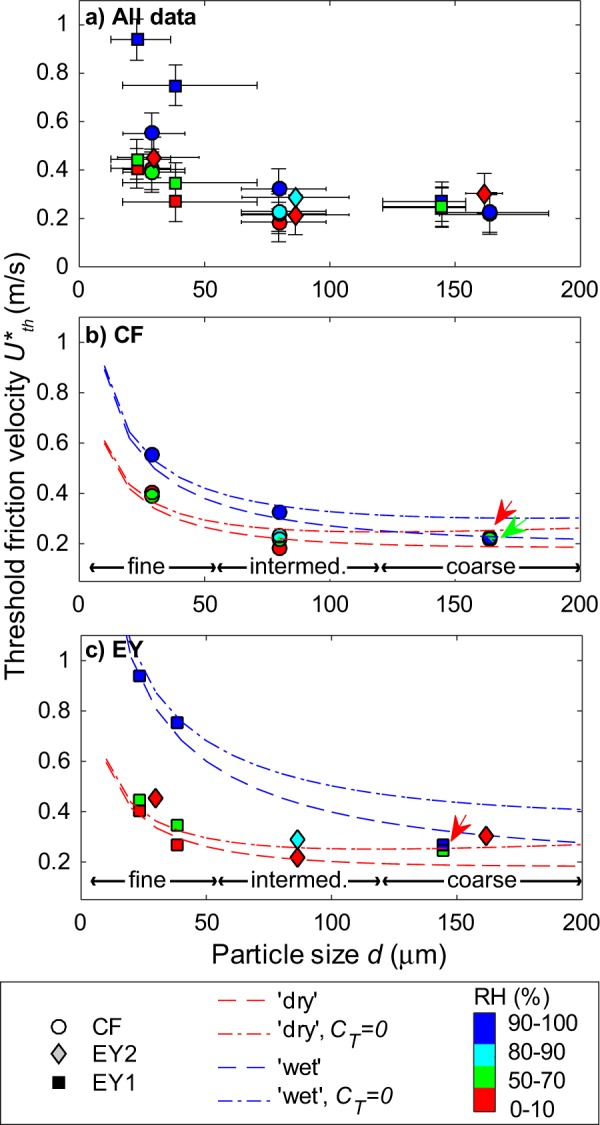
Measured threshold friction velocity U*_th_ (error of ±0.08 m/s, see text) for the different diameters *d* (median of each size distribution, with error bars marking the 25^th^ to the 75^th^ percentile range) for the *CF* and the two *EY* samples. The data are colour-coded by humidity range (RH) (**a**). Fit to the *CF* (**b)** and *EY* (**c**) data is provided by the Eq. 1 using fixed *C*_*T*_ and *C*_*L*_ coefficients for the minimum (red) and the maximum (blue) humidity ranges. The dash-dotted lines are for *C*_*T*_ = 0, where Eq. 1 equals the model of Shao and Lu^16^. The dashed lines are for *C*_*T*_ = 1.6 × 10^6^ m^−1^. Coloured arrows indicate overlapping data points.

*U*_th_* rapidly decreases as the median grain size of samples increases from 25 μm ca. to 80 μm ca., and then remains constant or slightly increases with increasing median size (Fig. 5a). The effect of RH on *U*_th_* display marked differences as a function of particle size and sample type. For the coarsest particles, *U*_th_* values fall in a very limited range (0.2–0.3 m/s) irrespectively of both RH and sample type. At intermediate particle size (median ca. 80 μm), *U*_th_* values at RH 90–100% are higher - albeit close to error bars - than those at RH 0–10%, and there is a small difference between the *CF* and *EY* samples. For the finest particles, the behaviour of the two samples is remarkably different, and *U*_th_* increases by a factor of 1.5 and 2.3 for *CF* and *EY* samples, respectively, when passing from the ‘dry’ to the ‘wet’ conditions (Fig. 5b,c).

The experimental data were fitted by the force balance model (Eq. 1)^21^

1 $${U}_{th}^{\ast }={(\frac{{\tau }_{th}}{{\rho }_{f}})}^{0.5}={(\frac{\frac{4}{3}\pi g\rho {r}^{3}+2{C}_{adh}r}{{C}_{L}{r}^{2}+{C}_{T}{r}^{3}}\cdot \frac{1}{{\rho }_{f}})}^{0.5}$$

where *τ*_*th*_ is the threshold shear stress, *r* is particle radius (equal to *d*/2), *g* is gravity, *ρ* is particle density, *ρ*_*f*_ is fluid density (1.22 kg/m^3^) and *C*_*adh*_ (N/m), *C*_*L*_ (dimensionless), and *C*_*T*_ (m^−1^) are the adhesion, lift, and torque coefficients of Merrison *et al*.^21^, respectively (see Supplementary Note).

We modelled separately the *CF* and *EY* sample data, considering only the RH 0–10% and the RH 90–100% cases, using fixed values for the *C*_*L*_ and *C*_*T*_ coefficients. In a first case, we used *C*_*L*_ = 160 and *C*_*T*_ = 0, in which case the model in Eq. 1 equals that of Shao and Lu^16^. In a second case, *C*_*T*_ was fixed at 1.6 × 10^6^ m^−1^, in agreement with previous experiments^21^ and models (Merrison, J. *et al*. Laboratory studies of the resuspension of volcanic dust by wind. Submitted to *J*. *Aerosol Sci*.) (Fig. 5b,c, Table 1).

**Table 1:** Fitting coefficients used for modelling the experimental data with Eq. 1.

Coefficients	*CF* RH 0–10%	*EY* RH 0–10%	*CF* RH 90–100%	*EY* RH 90–100%
*C*_*adh*_ (N/m)	1.8 × 10^−4^	1.8 × 10^−4^	4.0 × 10^−4^	1.1 × 10^−3^
*C*_*L*_ (adim.)	160	160	160	160
*C*_*T*_ (m^−1^)	0 or 1.6 × 10^6^	0 or 1.6 × 10^6^	0 or 1.6 × 10^6^	0 or 1.6 × 10^6^
*ρ* (kg/m^3^)	2384	2659	2384	2659

*C*_*T*_ and *C*_*L*_ coefficients were fixed either according to Merrison *et al*.^21^ or, neglecting *C*_*T*_, effectively rendering Eq. 1 equal to the Shao and Lu^16^ model. The *C*_*adh*_ coefficient are the best fit values. The value of *ρ* is the average density of the particles in the three size classes, including vesicularity.

Setting *C*_*T*_ = 0 m^−1^, provides better fit to the ‘dry’ data with respect to setting *C*_*T*_ = 1.6 × 10^6^ m^−1^. The opposite is true for the ‘wet’ data (Fig. 5b,c). *C*_*adh*_ values increase by a factor of between 2 and 6 when passing from the RH 0–10% to the RH 90–100% ranges.

## Discussion

Under dry environmental conditions, the resuspension threshold for volcanic ash as a function of particle size was observed to be in reasonable agreement in both magnitude and trend to previous experimental studies of volcanic and non-volcanic particles^21,25,32^ and also in reasonable agreement with expected values from resuspension models^16,17,21^. For instance, our results confirm the occurrence of the resuspension ‘optimum’ for dry volcanic particles around 80 μm in size, as previously recognised for non-volcanic particles^15^, as the result of the balance between adhesion and gravity forces^22^.

Under humid environmental conditions, the increase of *U*_th_* with increasing RH appears to be strongly non-linear, with only minor changes until high RH values. Previous experiments on synthetic particles found a critical RH around 60%, dependent upon material properties, above which capillary forces start playing a role and below which adhesion forces are constant^29^. At our experimental conditions, a critical RH value exists also for volcanic ash, constrained between 70–100% and 90–100% RH, for the fine and intermediate particles respectively. Above this RH range, the moisture content of our ‘wet’ samples may be sufficient to lead to the formation of (at least) a monolayer of water covering individual particles^19^, in line with the above findings. The intervening of capillary forces may also explain why, in the investigated particle size range, there is no evident resuspension ‘optimum’ for the ‘wet’ cases. Residence time of particles in a wet environment has an effect on hindering resuspension^24^. In this regards, the only constraints we can provide come from the dry *CF* sample that was run at ‘wet’ conditions (Fig. 4a). Results show that a few minutes in a ‘wet’ environment are not sufficient to change the resuspension behaviour, possibly because such a time interval is not enough to allow for the nucleation and growth of liquid water between the particles. This observation may have implications for the resuspension of freshly deposited ash during ongoing eruptions.

The ‘force balance’ model^21^ effectively reproduces the experimental data. In particular, all of our experimental variability can be explained by changes in the adhesion coefficient while leaving the torque and lift coefficients constant and in line with literature ranges (Table 1). The adhesion forces that we found are within the literature range for the dry cases^32^, while showing an increase in particle adhesion by a factor of between 2 and 6 (Table 1) in the presence of high humidity. This increased adhesion with humidity would be expected due to water bridging (i.e. capillary forces, function of particle gap and amount of liquid water^26^) and in fact is a moderate increase compared to previous observations of the dependence of particle adhesion on humidity for artificial particles^28^.

The observed increase in resuspension threshold at high humidity is strongly reduced down to negligible values for the larger particles (size class 125–250 µm). This point to a size dependence in the humidity-induced adhesion, possibly as a consequence of decreasing surface to mass ratio with increasing particle size. It is also possible that the higher vesicularity of the larger particles is reducing the amount of water available for bonding (see below). The best fit for the ‘dry’ and ‘wet’ cases with *C*_*T*_ = 0 m^−1^ and *C*_*T*_ = 1.6 × 10^6^ m^−1^, respectively, may reflect this trend, implying that rolling is an important factor only in the ‘wet’ cases. Further work would be needed here to confirm this observation.

Strikingly, there is a large difference in the effect of high humidity on the resuspension threshold between the *CF* and *EY* ash samples, with *U*_th_* increasing significantly more for *EY* than for *CF* for samples with grain size ranging 20–40 μm (Fig. 5b,c). The *U*_th_* = 0.95 m/s of the finest, ‘wet’ *EY* sample is almost double the value of the corresponding *CF* sample, despite the similar shape factors values and absence of secondary minerals in both samples. The observed difference could originated from the different vesicularity of the two samples (Fig. 3). The slightly higher vesicularity of the *CF* samples could result, for a similar amount of water in the sample, in a larger amount of water entering the particle vesicles and thus less water being available to form inter-particle bonding films.

**Figure 6:**
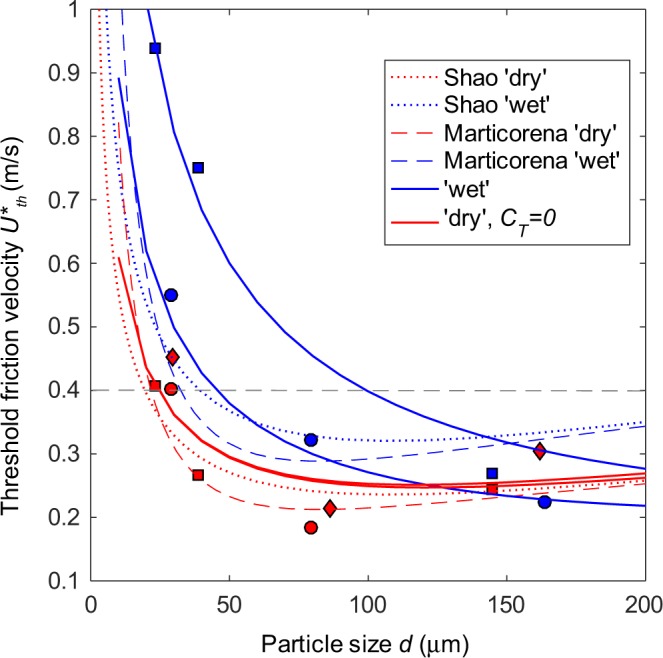
Comparison between: i) our ‘wet’ and ‘dry’ experimental data points (symbols as per Fig. 5), ii) our model fits (Eq. 1) with coefficients from Table 1 (solid lines), and iii) literature models^16,17^(dashed and dotted lines) with parameters previously used for the resuspension of volcanic ash particles by Folch *et al*.^8^. For sake of visibility, literature models are only shown for the *CF* sample, differences between the two samples being negligible. The grey dashed line marks the *U*_th_* = 0.4 m/s value previously used to model Eyjafjallajökull resuspension events^11,12,14^.

In some previous models of volcanic ash resuspension events at Eyjafjallajökull volcano, a constant *U*_th_* value of 0.4 m/s has been assumed regardless of ash size and environmental conditions^11,12,14^. This choice, although operationally straightforward, is suitable for dry fine ash only, but leads to underestimating the amount of resuspended coarser particles and largely overestimating that of fine wet ash (*U*_th_* is by a factor >2 higher in the EY <63 case, Fig. 6). In other cases^8^, existing size dependent models of *U*_th_*^16,17^ have been used, also implemented for the effect of moisture^26^. In order to compare these previous models with those which have been utilised here (Eq. 1), they have also been applied to our data sets using the same parameter values of Folch *et al*.^8^ but with the appropriate values for particle density (Table 1) and gravimetric soil moisture (0.6 wt.% with no adsorbed water).

For the ‘dry’ data, all the models, previous and current, provide results that are similar and congruent, irrespective of the sample type. The ‘Marticorena’ model^17^ seems to capture better than the ‘Shao’ model^16^ the ‘resuspension optimum’ around the 80 μm particle size. The ‘force balance’ model with *C*_*T*_ = 0 provides adhesion coefficient values almost identical to those assumed in Folch *et al*.^8^ for the ‘Shao’ model^16^ (Fig. 6). We conclude that, in first approximation, the resuspension of volcanic ash particles in dry environmental conditions can be modelled by using Eq. 1 and assuming *C*_*L*_ = 160, *C*_*T*_ = 0 m^−1^, and *C*_*adh*_ of 1.8 × 10^−4^ N/m, irrespectively of the specific particle features. Although we did not model the intermediate RH values samples, they almost overlap with the ‘dry’ ones (Fig. 5).

For the ‘wet’ data, literature models used a simple correction factor to increase *U*_th_* in the presence of atmospheric humidity (and enhanced moisture content of the ash), resulting in parallel trends of the ‘dry’ and ‘wet’ curves of *U*_th_* vs. particle grain size^8^. These modelling conditions fail to capture the size-dependent effect of humidity seen in the data, where *U*_th_* values converge towards the ‘dry’ ones as particle grain size approaches the coarsest size class. In the ‘wet’ case, we support the use of the ‘force balance’ model with *C*_*L*_ = 160, *C*_*T*_ = 1.6 × 10^6^ m^−1^, and *C*_*adh*_ of 4.0 × 10^−4^ and 1.1 × 10^−3^ N/m for the resuspension of *CF* and *EY* ash deposits, respectively.

In conclusion, these first wind tunnel experiments on the resuspension of volcanic ash at controlled environmental humidity highlight some peculiarities with respect to non-volcanic materials. Two main outcomes emerge. First, high humidity effectively hinders resuspension only for particles smaller than about fifty microns, possibly as the result of their smaller vesicularity. Second, resuspension behaviour may be largely different for ash deposits originated from different eruptions (i.e., with different particle properties and hence water bridging conditions), but only above a critical environmental humidity. Ash from the Eyjafjallajökull eruption is increasingly harder to remobilise than that of the Campi Flegrei one as the grain size decreases, reaching a factor of two in threshold friction velocity for particles smaller than about fifty microns. We point to the overall effectiveness of the ‘force balance’ model in capturing and offering a relatively simple physical explanation to the observed peculiarities. These peculiarities must be accounted for in future modelling and in hazard forecast preparedness concerning ash resuspension events. Further experimental studies are planned to investigate: i) micro-scale detachment dynamics, also in relation with the formation/disruption of ash aggregates^41^; and ii) changes in *U*_th_* as a function of the residence time and complex grain size distributions of ash layers.

## Electronic supplementary material

Supplementary Note
