# Supplementary material for: Experimental simulations of volcanic ash resuspension by wind under the effects of atmospheric humidity
Source: Sci Rep. 2018 Sep 28;8:14509. doi: 10.1038/s41598-018-32807-2 (PMC6162286; doi:10.1038/s41598-018-32807-2)
Supplement: Supplementary file 1 — Supplementary Note [file 41598_2018_32807_MOESM1_ESM.docx]

**Supplementary Note for**

**Experimental simulations of volcanic ash resuspension by wind under the effects of atmospheric humidity**

E. Del Bello*^1^, J. Taddeucci^1^, J. P. Merrison^2^, S. Alois^2^, J. J. Iversen^2^, P. Scarlato^1^

^1^ Istituto Nazionale di Geofisica e Vulcanologia, Sezione Roma1, Via di Vigna Murata 605, 00143 Roma, Italy.

^2^ Department of Physics and Astronomy, Aarhus University, Ny Munkegade 120, 8000, Aarhus C, Denmark.

*Corresponding author: Elisabetta Del Bello ([elisabetta.delbello@ingv.it)](mailto:elisabetta.delbello@ingv.it))

**Force balance model description**

In the simple force balance models applied here the threshold for wind induced particle resuspension is achieved when the lift and drag forces applied by the wind balance the gravitational and adhesive forces preventing detachment. Assumptions in force balance models are that the particles are spherical (with radius r) and that there exists a simple boundary layer flow profile with the forces exerted by the fluid (lift and drag) characterized by the friction velocity u* (i.e. the shear stress; $\tau=\rho{u_{*}}^{2}$). The particle size is assumed to be smaller than the viscous sub-layer.

In almost all such force balance expressions these forces are quantified based upon;

**Gravitational Force** = $\frac{4}{3}\pi g\rho r^{3}$

**Adhesive Force** = $2C_{adh}r$

Where Cadh is the adhesion coefficient (N/m)

**Lift Force** = $C_{L}r^{2}\tau$

Where C_L_ is the lift coefficient (dimensionless)

In the case of the modified model of Merrison et al.^1^ there is an additional drag induced rolling/sliding force (sometimes referred to as a torque force);

**Torque Force** = $C_{T}r^{3}\tau$

Where C_T_ is the drag induced torque coefficient (m^-1^)

Combining these expressions leads to Equation 1 for the threshold condition for detachment^1^ i.e.;

$$\tau_{det}=\frac{\frac{4}{3}\pi g\rho_{p}r^{3}+2C_{adh}r}{C_{L}r^{2}+C_{T}r^{3}}$$

In principle the parameters C_T_, C_L_ and C_adh_ are all empirically derived (free) values. However based upon previous works the parameter values found for dry Terrestrial conditions using the Shao and Lu^2^ model for sand are approximately; C_T_=0 m^-1^, C_L_=160, C_adh_=1.5x10^-4^ N/m.

In this study the humidity dependent parameter is assumed to be the adhesive force (adhesion coefficient). Quantifying the expected adhesion force for a 100µm particle for example gives a value in the case of dry material of around; Fadh≈1.5nN. In the case of increased humidity the C_adh_ parameter is increased in order to best fit the observed data.

C_T_ is also a free parameter which is presumably dependent upon the ability of the particles to roll (or slide) under drag induced forces, this has no apparent humidity dependence.

**References**

1. Merrison, J. P., Gunnlaugsson, H. P., Nørnberg, P., Jensen, A. E. & Rasmussen, K. R. Determination of the wind induced detachment threshold for granular material on Mars using wind tunnel simulations. *Icarus* **191,** 568–580 (2007).

2. Shao, Y. & Lu, H. A simple expression for wind erosion threshold friction velocity. *J. Geophys. Res.* **105,** 22437 (2000).
